# LGM2605 Reduces Space Radiation-Induced NLRP3 Inflammasome Activation and Damage in In Vitro Lung Vascular Networks

**DOI:** 10.3390/ijms20010176

**Published:** 2019-01-05

**Authors:** Shampa Chatterjee, Ralph A. Pietrofesa, Kyewon Park, Jian-Qin Tao, Alejandro Carabe-Fernandez, Abigail T. Berman, Constantinos Koumenis, Thais Sielecki, Melpo Christofidou-Solomidou

**Affiliations:** 1 Department of Physiology, University of Pennsylvania Perelman School of Medicine, Philadelphia, PA 19104, USA; shampac@pennmedicine.upenn.edu (S.C.); taoj@pennmedicine.upenn.edu (J.-Q.T.); 2 Division of Pulmonary, Allergy, and Critical Care, Department of Medicine, University of Pennsylvania Perelman School of Medicine, Philadelphia, PA 19104, USA; ralphp@pennmedicine.upenn.edu (R.A.P.); kyewpark@pennmedicine.upenn.edu (K.P.); 3 Department of Radiation Oncology, University of Pennsylvania Perelman School of Medicine, Philadelphia, PA 19104, USA; a.carabe@pennmedicine.upenn.edu (A.C.-F.); abigail.berman@uphs.upenn.edu (A.T.B.); koumenis@upenn.edu (C.K.); 4 LignaMed LLC, Philadelphia, PA 19104, USA; thais.sielecki@lignamed.com

**Keywords:** antioxidant, inflammasome, LGM2605, lignan, oxidative stress, phase II enzymes, reactive oxygen species, secoisolariciresinol diglucoside, space radiation

## Abstract

Updated measurements of charged particle fluxes during the transit from Earth to Mars as well as on site measurements by Curiosity of Martian surface radiation fluxes identified potential health hazards associated with radiation exposure for human space missions. Designing mitigation strategies of radiation risks to astronauts is critical. We investigated radiation-induced endothelial cell damage and its mitigation by LGM2605, a radioprotector with antioxidant and free radical scavenging properties. We used an in vitro model of lung vascular networks (flow-adapted endothelial cells; FAECs), exposed to gamma rays, low/higher linear energy transfer (LET) protons (3–4 or 8–10 keV/µm, respectively), and mixed field radiation sources (gamma and protons), given at mission-relevant doses (0.25 gray (Gy)–1 Gy). We evaluated endothelial inflammatory phenotype, NLRP3 inflammasome activation, and oxidative cell injury. LGM2605 (100 µM) was added 30 min post radiation exposure and gene expression changes evaluated 24 h later. Radiation induced a robust increase in mRNA levels of antioxidant enzymes post 0.25 Gy and 0.5 Gy gamma radiation, which was significantly decreased by LGM2605. Intercellular cell adhesion molecule-1 (ICAM-1) and NOD-like receptor protein 3 (NLRP3) induction by individual or mixed-field exposures were also significantly blunted by LGM2605. We conclude that LGM2605 is a likely candidate to reduce tissue damage from space-relevant radiation exposure.

## 1. Introduction

Updated measurements of charged particle fluxes during the transit from Earth to Mars conducted with the Radiation Assessment Detector inside the Mars Science Laboratory spacecraft [1], as well as on site measurements by Curiosity of Martian surface radiation fluxes [2,3], provided valuable knowledge for a better evaluation of potential health hazards associated with human space missions [4,5]. While space travel opens the door to numerous exciting and fascinating possibilities, it is associated with such risks that impact the crewmember’s health and performance, thus jeopardizing the success of future missions to distant destinations. Therefore, designing mitigation of risks to astronauts, such as those from exposure to space radiation during transit and after landing/colonization, is an unmet need. Importantly, potentially life-threatening risks of developing cancer, brain and central nervous system damage, and other tissue degenerations have been identified that result from radiation exposure unique to space travel. Two forms of radiation pose potential health risks to astronauts in deep space. There is a chronic low-dose exposure to galactic cosmic rays (GCRs), as well as the possibility of short-term exposures to the solar energetic particles (SEPs) that are sporadically accelerated close to the Sun by solar flares and coronal mass ejections or solar particle events (SPEs). GCRs tend to be highly energetic; highly penetrating particles that are not stopped by the modest depths of shielding on a typical spacecraft.

Adverse radiation effects associated with space travel include damage to the immune system, the central nervous system [6], and the lens of the eye [7], along with potential cancer development [8]. Oxidative damage to cells and biomolecules is directly associated with the severity of damage from space radiation exposure [9]. Risk estimates have been developed for incidence and mortality of cancers, like leukemia and solid tumor cancers [10]. However, when considering the lifetime total radiation risk (TRR) of astronauts for space flights, it has been shown that TRR in senior-age astronaut groups is more often due to non-tumor death [11], including vascular changes and blood circulation abnormalities. Similarly, epidemiological data have shown that A-bomb survivors show increased non-cancer mortality due to cardiovascular injury [12]. However, vascular signaling and damage in response to space relevant radiation has never been studied. Additionally, among the organs, radiation poses a severe health risk to the respiratory system and the lungs [13]; being an interface between air and tissue, and a major target of space radiation exposure. We thus, propose to evaluate space radiation-induced signaling in an in vitro model of the pulmonary vasculature that mimics the vascular network in vivo and use this model to test a potential medical countermeasure agent.

The vascular system, a conduit and connector of blood flow across organs, is a network that integrates biochemical and biophysical signals via systemic transport of blood, nutrients, and inflammatory, pathogenic moieties across the body. In addition, the vasculature is an initiator and converging site of inflammation, a pivotal event in organ injury. Initiation of inflammation occurs via production of moieties that facilitate recruitment and adherence of immune cells (neutrophils, macrophages, and leukocytes) to the vascular wall. Adherence of immune cells is followed by extravasation of these cells into tissue, where these cells release large amounts of radicals (such as reactive oxygen species and peroxyl radicals) that damage tissue. The vasculature is thus, increasingly being recognized as an important target with radiation exposure; indeed, vascular damage upon radiation exposure has been well documented [14,15,16] and hemorrhage and injury to the vasculature, as well as circulatory problems, have been recognized as a major cause of death. Space radiation effects on the vasculature, in terms of high incidence of cardiovascular disease mortality on astronauts, have been reported [17]. Furthermore, exposure of mice to silicon (0.5 Gy) or iron (0.15 Gy) ions has been reported to induce infiltration of immune cells, as well as to increase pro-inflammatory cytokines [5,18]. These are indicative of an inflammatory response, and as endothelial cells are the primary cells of the vasculature and both the initiators and site of inflammation, understanding space radiation-induced endothelial signaling that leads to eventual vascular damage is very pertinent. Our goal is to determine the effects of space-relevant radiation by identifying the endothelial cell (EC) signaling that occurs in a space-relevant radiation regimen, so as to block these early signals and minimize downstream events that progress to cause severe endovascular injury and hemorrhage. We selected to use low dose gamma since it is often used as a surrogate to proton radiation which constitutes ~85% of the GCR in open space, it is reflected from the surface of Mars [2] and is also known to be a source of oxidative stress and damage and the bystander product of proton degradation [19]. In addition, we tested low linear energy transfer (LET) proton, high LET proton, and mixed radiation exposures of gamma and protons.

We have identified whole grain flaxseed (FS) and its bioactive lignan component (FLC) as potent protectors against radiation-induced lung injury [20,21]. Specifically, FS as well as FLC enriched in the phenolic, secoisolariciresinol diglucoside (SDG) given after thoracic radiation mitigated radiation effects by decreasing pulmonary fibrosis, inflammation, and cytokine release, while improving blood oxygenation levels and overall mouse survival [20]. In further studies using space-relevant radiation (proton radiation), we have shown that LGM2605 (the synthetic version of SDG) acts as a mitigator of radiation-induced increases in inflammatory signals (cytokines, chemokines) in human lung sections (huPCLS) [22]. LGM2605 is a potent scavenger of radiation-induced reactive oxygen species (ROS) and active chlorine species (ACS), and can reduce the ionizing species arising from radiation [23,24,25,26]. However, more importantly, it is also an inhibitor of several proinflammatory signaling pathways that upregulate cytokines, chemokines, and adhesion molecules [24,27].

Space radiation-induced injury to various cells and tissues has been studied in the past; indeed, endothelial cells in culture have been examined for space radiation induced damage. However, models that closely represent the vasculature in vivo (which is unique by virtue of being an integrative snapshot of the “health” of the vertebrate) have never been used. We therefore examined space radiation effects on a model of flow adapted endothelial cells to mimic the vasculature in vivo. Our goal of evaluating space radiation-induced signaling that is injurious to the vascular network is a first step in protection against inflammation and vascular disease that may arise from exposures associated with deep space missions. Thus, we utilized a model of the lung vascular network “in vivo” [28]. Since the vasculature is exposed to constant blood flow, which alters the vascular phenotype, we reasoned that using endothelial cells adapted to flow would be representative of the vascular network. Such cells, as we have previously reported, respond similarly to endothelial cells on the vascular bed of a “live” lung [29,30,31]. Thus, we used the flow-adapted endothelial cell (FAEC) model where endothelial cells “face” blood flow in parallel plate chambers while being exposed to radiation; thus, recapitulating the space radiation exposure of astronauts. FAECs were exposed to low dose gamma rays, low LET protons (3–4 keV/µm), higher LET protons (8–10 keV/µm), and mixed field radiation sources (gamma and protons), given at mission-relevant doses (0.25–1 Gy) to mimic space-relevant radiation species used in many of the previously-cited studies [32,33]. Gamma and proton radiation are recognized as potent generators of tissue damage, whereby gamma rays are often used as surrogate to proton radiation which constitutes approximately 85% of the GCR in open space [2]. Gamma radiation is also reflected from the surface of Mars [2]. We aimed to characterize the damaging effects of space-relevant radiation exposure on the vasculature in terms of endothelial cell inflammatory phenotype as assessed by induction of intercellular adhesion molecule (ICAM-1) and NLRP3 inflammasome induction and activation, and oxidative injury [30,34,35,36,37]. Using this in vitro model of lung vascular networks (FAEC model), we were able to evaluate the radiation mitigating properties of LGM2605.

## 2. Results

We first validated the in vitro lung vascular network cell system as a model to study space radiation damage to the endovasculature at 24 h post exposure of FAECs to low dose gamma (see Section 2.1), low LET (3–4 keV/µm) proton radiation (see Section 2.2), high LET (8–10 keV/µm) proton radiation (see Section 2.3), or mixed field gamma and proton radiation exposure (see Section 2.4) by evaluating induction of the endothelial cell inflammatory phenotype (ICAM-1) and NLRP3 inflammasome induction and activation. In order to determine whether LGM2605, given post radiation exposure, is an effective mitigator of space radiation-induced vascular damage, we evaluated the dampening of the endothelial inflammatory phenotype and its functional effects, and NLRP3 inflammasome activation. In the subsequent studies, we exposed FAECs to gamma radiation, low LET proton radiation, high LET proton radiation, or mixed field radiation. LGM2605 was added 30 min post exposure and cells were evaluated 24 h later for expression of the cell adhesion molecule ICAM-1, a known marker of the inflammatory phenotype of cells [35,38], and NLRP3 inflammasome activation.

### 2.1. LGM2605 Alters the Antioxidant Response in In Vitro Lung Vascular Networks and Mitigates Gamma Radiation-Induced Inflammatory Phenotype

**Scheme 1:**
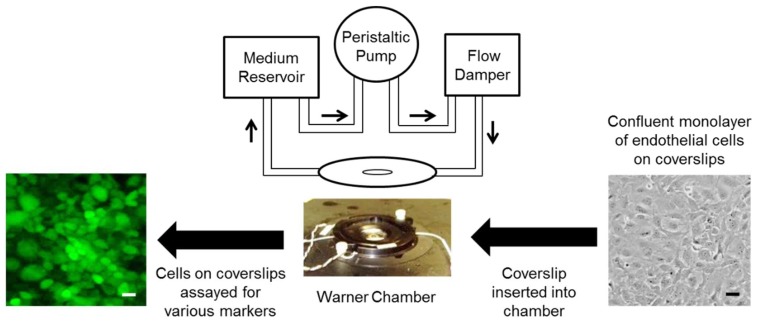
The vascular network as represented by flow adapted endothelial cells (FAECs). Since the vascular network is exposed to blood flow in vivo, any in vitro model that recapitulates the vasculature requires that endothelial cells be kept under flow. Here endothelial cells grown on coverslips are inserted into parallel plate chamber (Warner Instruments, LLC, Hamden, CT, USA) and attached into a perfusion circuit. The perfusate from the reservoir is drawn by the peristaltic pump into the flow chamber (via a second reservoir or flow damper). The shear stress generated is ~7 dyn/cm^2^, which is representative of the shear associated with blood flow in capillaries [39]. After 24 h of flow adaptation, these cells are exposed to space relevant radiation and assayed for various markers. Scale bar = 25 μm.

The in vitro lung vascular network system involves “flow-adapted” endothelial cells (FAECs), which simulates the endothelium in vivo where cells “face” blood flow. FAECs allow us to evaluate the responses of an endothelial monolayer alone (without confounding contributions from other cell types) to radiation exposure. Cells are kept under flow in either parallel plate chambers for 24 h [29,30]. FAECs on the coverslips allow for immunostaining of endothelial inflammatory moieties such as ICAM-1 and NLRP3 subunit of the inflammasome (Scheme 1).

**Figure 1:**
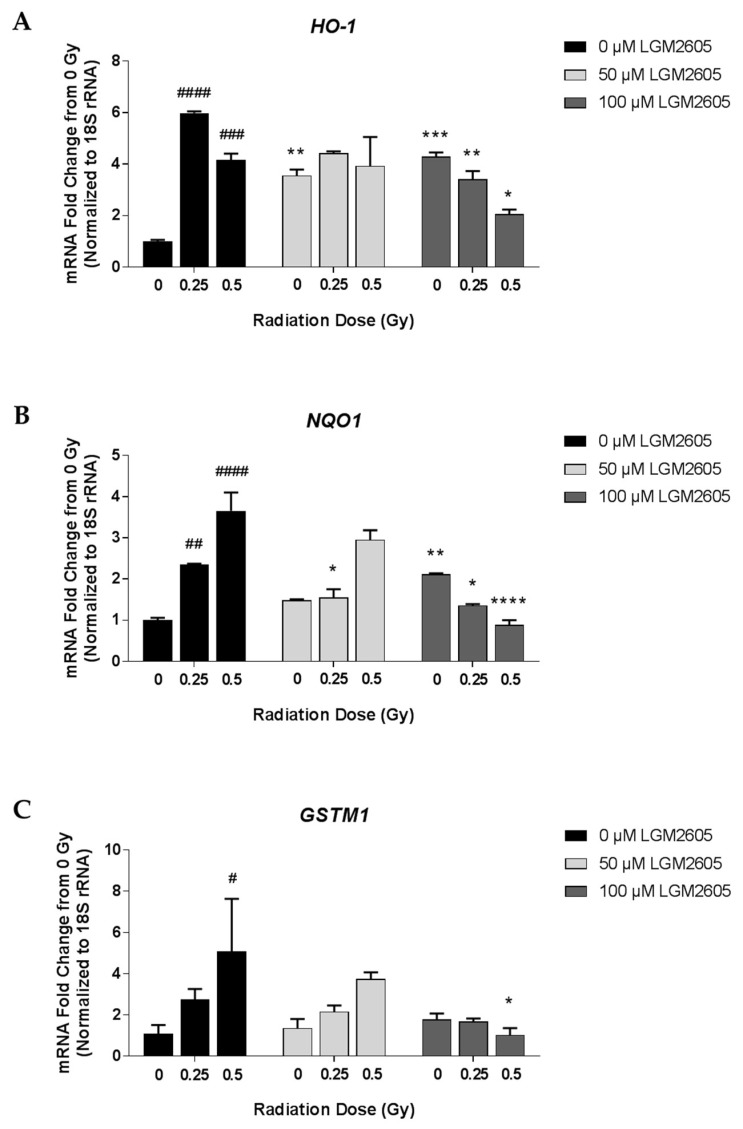
Determination of the antioxidant response following gamma radiation exposure of in vitro lung vascular networks (flow-adapted endothelial cells) and LGM2605 (synthetic secoisolariciresinol diglucoside (SDG)) treatment. FAECs were exposed to 0 Gy, 0.25 Gy, or 0.5 Gy gamma radiation and treated with 0 µM, 50 µM, or 100 µM LGM2605 30 min following radiation exposure. Cells were harvested 24 h post radiation exposure and mRNA expression of *HO-1* (**A**), *NQO1* (**B**), and *GSTM1* (**C**) was determined using qPCR. Levels of target gene mRNA were normalized to 18S ribosomal RNA and the values are expressed as mean fold change from FAECs exposed to 0 Gy and treated with 0 µM LGM2605. Data are presented as mean ± SEM. # shown in figures indicate significant differences from FAECs exposed to 0 Gy gamma radiation and treated with 0 µM LGM2605 (# *p* < 0.05; ## *p* < 0.01; ### *p* < 0.001; #### *p* < 0.0001). Asterisks shown in figures indicate significant differences from FAECs exposed to 0 Gy, 0.25 Gy, or 0.5 Gy gamma radiation and treated with 0 µM LGM2605, respectively (* *p* < 0.05; ** *p* < 0.01; *** *p* < 0.001; **** *p* < 0.0001).

**Scheme 2:**
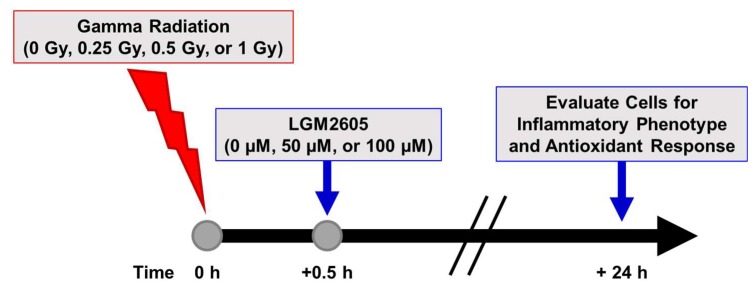
Experimental plan of gamma radiation exposure of in vitro lung vascular networks (flow-adapted endothelial cells) and LGM2605 treatment. FAECs were exposed to 0 Gy, 0.25 Gy, 0.5 Gy, or 1 Gy gamma radiation and treated with 0 µM, 50 µM, or 100 µM LGM2605 30 min following radiation exposure. Cells were harvested 24 h post radiation exposure and evaluated for inflammation, inflammasome activation, and gene expression changes related to profibrogenic and antioxidant processes.

Using qPCR, we first determined gene expression changes relating to the antioxidant response in FAECs exposed to 0 Gy, 0.25 Gy, 0.5 Gy, or 1 Gy gamma radiation and evaluated 24 h later (Scheme 2). Exposure to 0.25 Gy and 0.5 Gy gamma radiation led to significant (*p* < 0.05) increases in antioxidant genes, *HO-1*, *NQO1*, and *GSTM1*, at 24 h post radiation exposure. Treatment with 100 µM LGM2605-only significantly increased mRNA levels of *HO-1* and *NQO1* (Figure 1). While levels of *HO-1* were 6.0- and 4.2-fold increased over non-irradiated control FAECs among cells exposed to 0.25 Gy and 0.5 Gy, respectively, treatment with 100 µM LGM2605 decreased *HO-1* mRNA levels to 3.4- and 2.1-fold, respectively. Pretreatment with 100 µM LGM2605-alone 4 h prior to radiation exposure led to significantly increased levels of HO-1 and NQO1 mRNA when compared to non-irradiated FAECs treated with vehicle. In addition to the increase in the mRNA expression of cytoprotective antioxidant enzymes, LGM2605 is able to directly scavenge radiation-induced free radicals, such as reactive oxygen species and active chlorine species, which may decrease the need for cellular antioxidant defenses in FAECs exposed to ionizing radiation.

Relative quantification of target gene mRNA levels is shown normalized to 18S rRNA (Figure 1). Concordant findings were observed when utilizing GAPDH as the reference gene to normalize the data (data not shown). For example, mRNA levels of NQO1 were significantly increased following gamma radiation exposure for both 18S rRNA (1.49- and 2.12-fold change from 0 Gy for 0.25 Gy and 0.5 Gy, respectively) and GAPDH (3.43- and 2.81-fold change from 0 Gy for 0.25 Gy and 0.5 Gy, respectively) data normalization. Regardless of the reference gene utilized, treatment with 100 µM LGM2605 significantly decreased NQO1 mRNA levels by approximately 60% (58.29% when normalized to 18S rRNA and 62.03%, when normalized to GAPDH).

**Figure 2:**
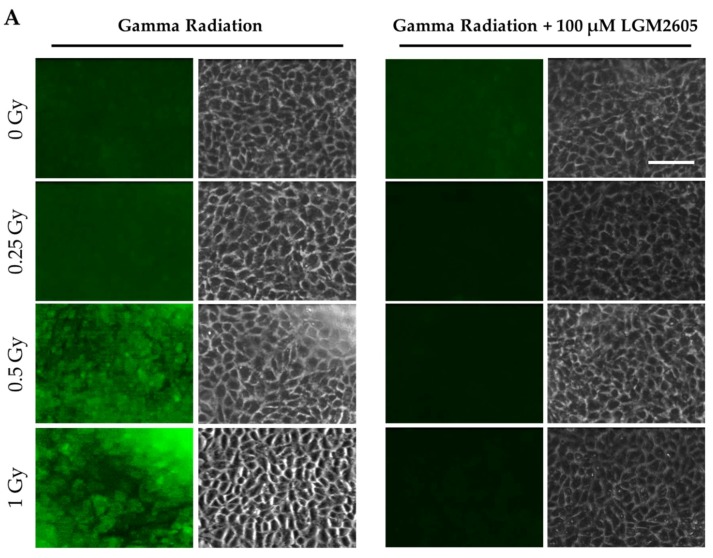

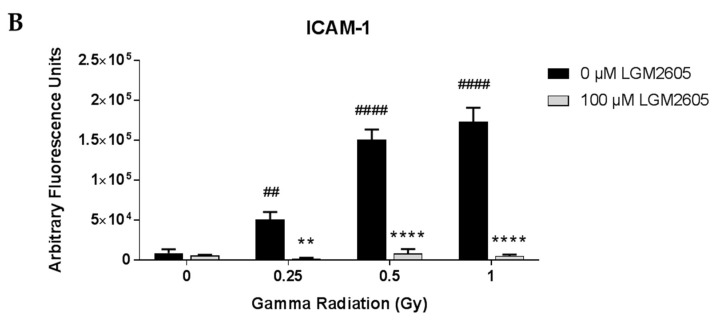
Evaluation of LGM2605 inhibition of intercellular cell adhesion molecule-1 (ICAM-1) expression in in vitro lung vascular networks (flow-adapted endothelial cells) exposed to gamma radiation. FAECs were exposed to 0 Gy, 0.25 Gy, 0.5 Gy, or 1 Gy gamma radiation and treated with 0 µM or 100 µM LGM2605 30 min following radiation exposure. Cells were harvested 24 h post radiation exposure and evaluated for ICAM-1 expression. Fluorescent images of FAECs exposed to LGM2605 (0 µM or 100 µM) 30 min following radiation exposure and harvested at 24 h radiation exposure and incubated with anti-ICAM-1 antibody (ThermoFisher Scientific, Waltham, MA, USA). Scale bar = 20 μm (**A**); graphed representation of the data imaged for each condition (**B**). Data are presented as mean ± SEM. # shown in figures indicate significant differences from FAECs exposed to 0 Gy gamma radiation and treated with 0 µM LGM2605 (## *p* < 0.01; #### *p* < 0.0001). Asterisks shown in figures indicate significant differences from FAECs exposed to 0.25 Gy, 0.5 Gy, or 1 Gy gamma radiation and treated with 0 µM LGM2605, respectively (** *p* < 0.01; **** *p* < 0.0001).

Expression of the cell adhesion molecule ICAM-1, a known marker of the inflammatory phenotype of cells, was also determined among FAECs exposed to gamma radiation (Figure 2). Importantly, exposure to gamma radiation led to a significant increase in ICAM-1 expression in a dose-dependent manner, which was significantly (*p* < 0.01) blunted by LGM2605 treatment administered 30 min post radiation exposure (97.1%, 94.5%, and 97.1% decrease by LGM2605 treatment among FAECs exposed to 0.25 Gy, 0.5 Gy, and 1 Gy gamma radiation, respectively).

### 2.2. LGM2605 Blunts Low LET Proton Radiation-Induced Inflammatory Phenotype in In Vitro Lung Vascular Networks

**Scheme 3:**
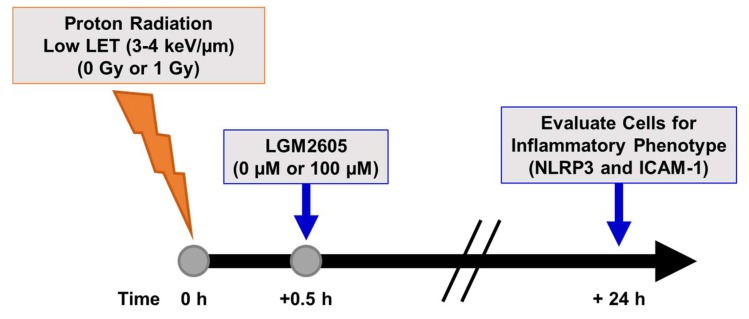
Experimental plan of low LET proton radiation exposure of in vitro lung vascular networks (flow-adapted endothelial cells) and LGM2605 treatment. FAECs were exposed to 0 Gy or 1 Gy low LET (3–4 keV/µm) proton radiation and treated with 0 µM or 100 µM LGM2605 30 min following radiation exposure. Cells were harvested 24 h post radiation exposure and evaluated for inflammation and inflammasome activation.

**Figure 3:**
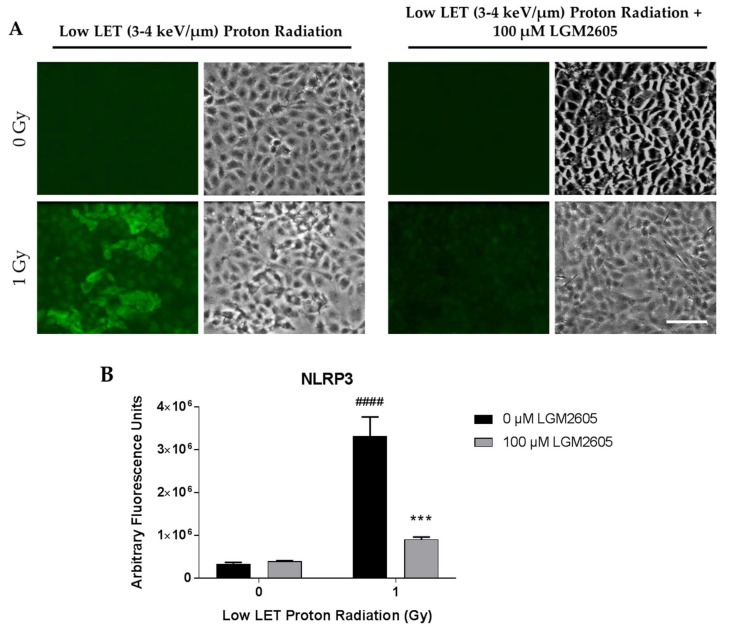
Evaluation of LGM2605 inhibition of NOD-like receptor protein 3 (NLRP3) activation in in vitro lung vascular networks (flow-adapted endothelial cells) exposed to low LET proton radiation. FAECs were exposed to 0 Gy or 1 Gy low LET (3–4 keV/µm) proton radiation and treated with 0 µM or 100 µM LGM2605 30 min following radiation exposure. Cells were harvested 24 h post radiation exposure and evaluated for NLRP3 inflammasome activation. Fluorescent images of FAECs exposed to LGM2605 (0 µM or 100 µM) 30 min following radiation exposure and harvested at 24 h radiation exposure and incubated with anti-NLRP3 antibody (R&D Systems, Minneapolis, MN, USA). Scale bar = 20 μm (**A**); graphed representation of the data imaged for each condition (**B**). Data are presented as mean ± SEM. # shown in figures indicate significant differences from FAECs exposed to 0 Gy low LET proton radiation and treated with 0 µM LGM2605 (#### *p* < 0.0001). Asterisks shown in figures indicate significant differences from FAECs exposed to 1 Gy low LET proton radiation and treated with 0 µM LGM2605 (*** *p* < 0.001).

**Figure 4:**
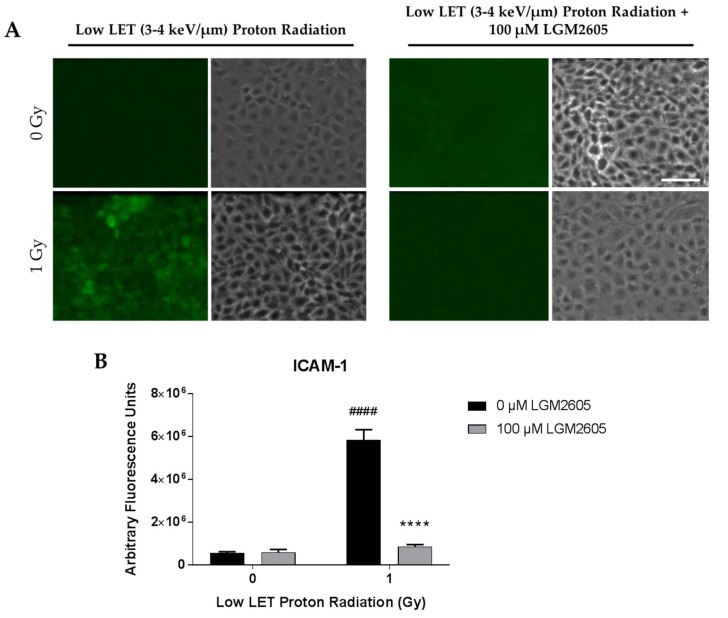
Evaluation of LGM2605 inhibition of ICAM-1 expression in in vitro lung vascular networks (flow-adapted endothelial cells) exposed to low LET proton radiation. FAECs were exposed to 0 Gy or 1 Gy low LET (3–4 keV/µm) proton radiation and treated with 0 µM or 100 µM LGM2605 30 min following radiation exposure. Cells were harvested 24 h post radiation exposure and evaluated for ICAM-1 expression. Fluorescent images of FAECs exposed to LGM2605 (0 µM or 100 µM) 30 min following radiation exposure and harvested at 24 h radiation exposure and incubated with anti-ICAM-1 antibody (ThermoFisher Scientific, Waltham, MA, USA). Scale bar = 20 μm (**A**); graphed representation of the data imaged for each condition (**B**). Data are presented as mean ± SEM. # shown in figures indicate significant differences from FAECs exposed to 0 Gy low LET proton radiation and treated with 0 µM LGM2605 (#### *p* < 0.0001). Asterisks shown in figures indicate significant differences from FAECs exposed to 1 Gy low LET proton radiation and treated with 0 µM LGM2605 (**** *p* < 0.0001).

We also determined the effects of low LET (3–4 keV/µm) proton radiation exposure in inducing the inflammatory phenotype and NLRP3 inflammasome activation in lung vascular networks. FAECs were exposed to 1 Gy low LET proton radiation and treated with 0 µM or 100 µM LGM2605 at 30 min post radiation exposure (Scheme 3). The pulmonary microvascular endothelial cells used in this study show a cobblestone pattern that does not change with flow adaptation. As we have reported earlier, these cells (unlike cells derived from large arteries or aorta which orient themselves in the direction of flow [40] do not show any phenotypical changes with long term exposure to flow. Upon exposure to gamma radiation and to low LET radiation, the cobblestone phenotype of these cells remained largely unaltered. FAEC expression of NLRP3 inflammasome was low among non-irradiated cells treated with or without 100 µM LGM2605. Exposure to 1 Gy low LET proton radiation significantly (*p* < 0.0001) increased NLRP3 levels. Treatment with LGM2605 30 min post radiation exposure blunted NLRP3 expression by 72.6% (Figure 3). Similarly, ICAM-1 expression was low among non-irradiated cells treated with or without 100 µM LGM2605. Exposure to 1 Gy low LET proton radiation significantly (*p* < 0.0001) increased ICAM-1 expression, which was ameliorated by LGM2605 treatment by 85.3% (Figure 4).

### 2.3. High LET Proton Radiation-Induced Increases in ICAM-1 and NLRP3 Are Mitigated by LGM2605 Treatment in In Vitro Lung Vascular Networks

**Figure 5:**
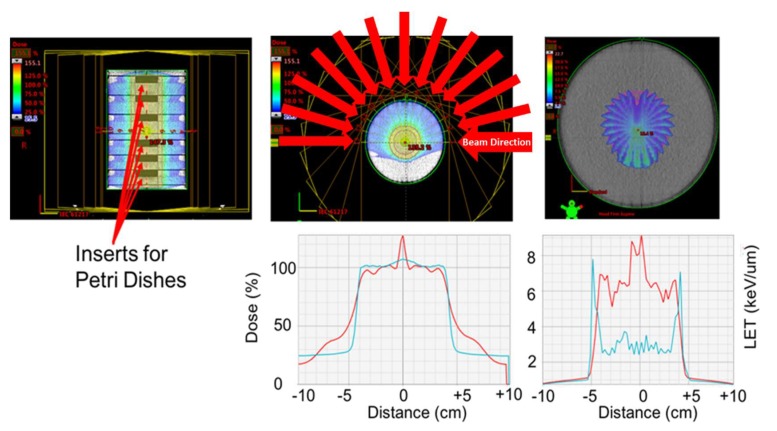
Geometry used to irradiate the cells with 0.5 and 0.25 Gy of protons, while maximizing the LET within the petri dishes. Top-left: CT image cross-section of the cylindrical phantom. The phantom is composed of six individual blocks made of solid water. Solid water phantoms are made of epoxy resins and powders to control density and radiation properties of the phantom and are traditionally used for dosimetry purposes. Each block was of 200 mm in diameter and 30 mm in depth. The blocks had a 60 mm insert centered in the central axis of the block, where the petri dishes were placed for the exposure. Each proton beam used had a vertical extension beyond the petri dish holder. Top-center: Color-wash of the proton dose distribution produced by 13 concentric and co-planar proton beams of 101 mm range. The 13 beams were delivered over an arc of 180 degrees and were separated every 15 degrees. Each beam only used a single energy of 117.14 MeV. Top-right: color-wash figure showing the Linear Energy Transfer distribution of the beam arrangement use. The image shows the LET increase at the distal falloff of each beam. Bottom-left: dose profile across the central plane of the cylinder (red line). The dose coverage extended 1 cm beyond the edges of the inserts to ensure the dishes received 100% of the dose. The blue line represents the dose profile that would be obtained if the beams had a Spread of Bragg Peak (SOBP), which improves the homogeneity of the dose. Bottom-right: Profile of the LET distribution across the target. The LET using the monoenergetic beams is higher within the Petri dishes, compared with the SOBP beams.

**Scheme 4:**
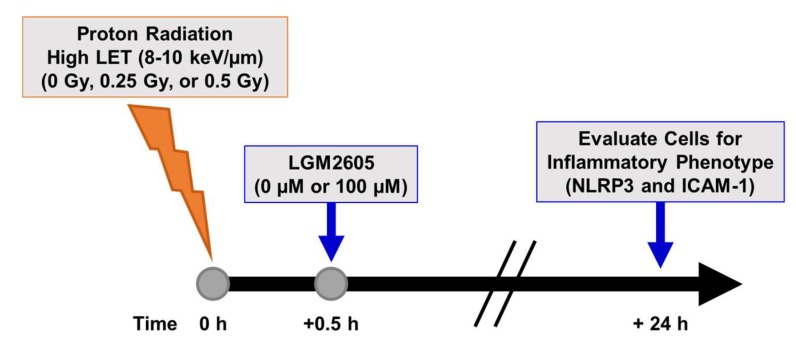
Experimental plan of high LET proton radiation exposure of in vitro lung vascular networks (flow-adapted endothelial cells) and LGM2605 treatment. FAECs were exposed to 0 Gy, 0.25 Gy, or 0.5 Gy high LET (8–10 keV/µm) proton radiation and treated with 0 µM or 100 µM LGM2605 30 min following radiation exposure. Cells were harvested 24 h post radiation exposure and evaluated for inflammation and inflammasome activation.

We also determined the effects of high LET (8–10 keV/µm) proton radiation exposure (see Figure 5) in inducing the inflammatory phenotype and inflammasome activation in lung vascular networks. FAECs were exposed to 0.25 Gy or 0.5 Gy high LET proton radiation and treated with 0 µM or 100 µM LGM2605 at 30 min post radiation exposure (Scheme 4).

**Figure 6:**
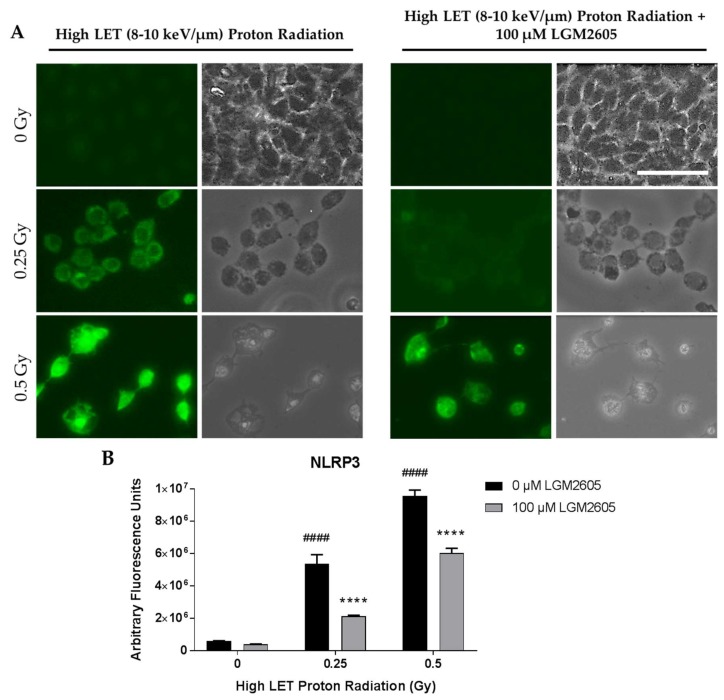
Evaluation of LGM2605 inhibition of NLRP3 activation in in vitro lung vascular networks (flow-adapted endothelial cells) exposed to high LET proton radiation. FAECs were exposed to 0 Gy, 0.25 Gy, or 0.5 Gy high LET (8–10 keV/µm) proton radiation and treated with 0 µM or 100 µM LGM2605 30 min following radiation exposure. Cells were harvested 24 h post radiation exposure and evaluated for NLRP3 inflammasome activation. Fluorescent images of FAECs exposed to LGM2605 (0 µM or 100 µM) 30 min following radiation exposure and harvested at 24 h radiation exposure and incubated with anti-NLRP3 antibody (R&D Systems, Minneapolis, MN, USA). Scale bar = 20 μm (**A**); graphed representation of the data imaged for each condition (**B**). Data are presented as mean ± SEM. # shown in figures indicate significant differences from FAECs exposed to 0 Gy high LET proton radiation and treated with 0 µM LGM2605 (#### *p* < 0.0001). Asterisks shown in figures indicate significant differences from FAECs exposed to 0.25 Gy or 0.5 Gy high LET proton radiation and treated with 0 µM LGM2605, respectively (**** *p* < 0.0001).

**Figure 7:**
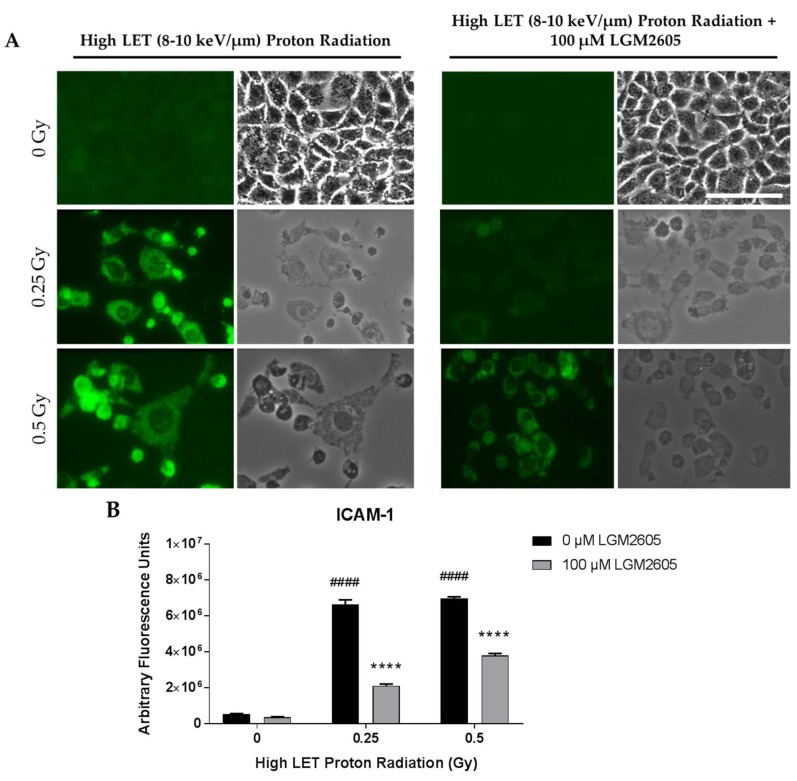
Evaluation of LGM2605 inhibition of ICAM-1 expression in in vitro lung vascular networks (flow-adapted endothelial cells) exposed to high LET proton radiation. FAECs were exposed to 0 Gy, 0.25 Gy, or 0.5 Gy high LET (8–10 keV/µm) proton radiation and treated with 0 µM or 100 µM LGM2605 30 min following radiation exposure. Cells were harvested 24 h post radiation exposure and evaluated for ICAM-1 expression. Fluorescent images of FAECs exposed to LGM2605 (0 µM or 100 µM) 30 min following radiation exposure and harvested at 24 h radiation exposure and incubated with anti-ICAM-1 antibody (ThermoFisher Scientific, Waltham, MA, USA). Scale bar = 20 μm (**A**); graphed representation of the data imaged for each condition (**B**). Data are presented as mean ± SEM. # shown in figures indicate significant differences from FAECs exposed to 0 Gy high LET proton radiation and treated with 0 µM LGM2605 (#### *p* < 0.0001). Asterisks shown in figures indicate significant differences from FAECs exposed to 0.25 Gy or 0.5 Gy high LET proton radiation and treated with 0 µM LGM2605, respectively (**** *p* < 0.0001).

FAEC expression of NLRP3 inflammasome was low among non-irradiated cells treated with or without 100 µM LGM2605. Exposure to 0.25 Gy or 0.5 Gy high LET proton radiation significantly (*p* < 0.0001) increased NLRP3 levels in a dose-dependent manner. Treatment with LGM2605 30 min post radiation exposure blunted NLRP3 expression by 60.5% and 37.0% among FAECs exposed to 0.25 Gy or 0.5 Gy high LET proton radiation, respectively (Figure 6). Similarly, ICAM-1 expression was low among non-irradiated cells treated with or without 100 µM LGM2605. Exposure to 0.25 Gy or 0.5 Gy high LET proton radiation significantly (*p* < 0.0001) increased ICAM-1 expression to equal levels. The observed increases in ICAM-1 expression following 0.25 Gy or 0.5 Gy high LET proton radiation exposure was ameliorated by LGM2605 treatment by 68.4% and 45.6%, respectively (Figure 7).

### 2.4. LGM2605 Ameliorates the Inflammatory Phenotype Induced by Mixed Field Gamma and Proton Radiation Exposure in In Vitro Lung Vascular Networks

**Scheme 5:**
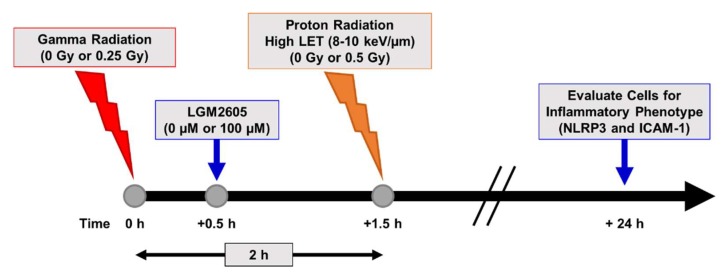
Experimental plan of mixed field radiation exposure of in vitro lung vascular networks (flow-adapted endothelial cells) and LGM2605 treatment. FAECs were exposed to 0 Gy or 0.25 Gy gamma radiation and subsequently exposed to 0 Gy or 0.5 Gy high LET (8–10 keV/µm) proton radiation. FAECs were treated with 0 µM or 100 µM LGM2605 30 min following gamma radiation exposure. Cells were harvested 24 h post radiation exposure and evaluated for inflammation and inflammasome activation.

Efficacy studies considering space-relevant exposures of biological structures such as cells and tissues, need to be evaluating not only individual radiation species, but also mixed field combinations. For this, we exposed FAECs to higher LET protons (8–10 keV/µm), which represent >60% of field contribution in space and combined this with 0.25 Gy gamma radiation exposure, totaling no more than 0.75 Gy total exposure. For the combined exposure, FAECs were exposed to 0.25 Gy gamma radiation and then exposed to high LET proton radiation 2 h later. LGM2605 treatment was initiated 30 min post gamma radiation exposure (Scheme 5).

**Figure 8:**
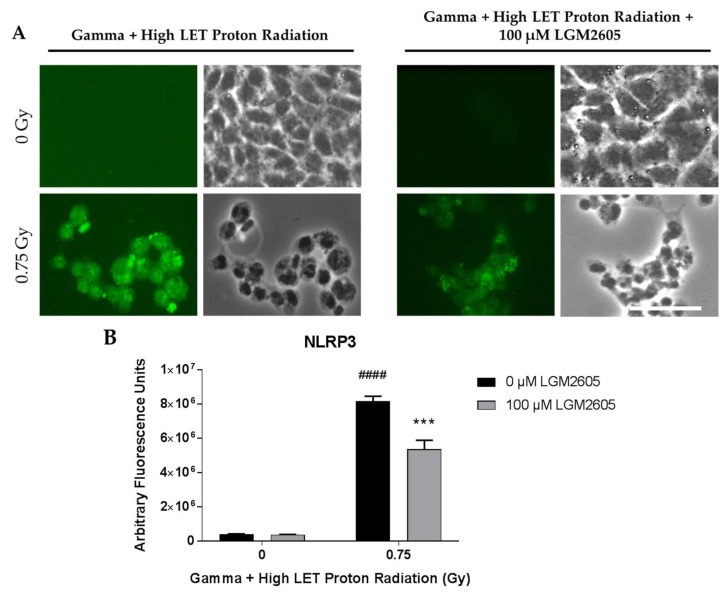
Evaluation of LGM2605 inhibition of NLRP3 activation in in vitro lung vascular networks (flow-adapted endothelial cells) exposed to mixed field gamma and proton radiation. FAECs were exposed to 0 Gy or 0.25 Gy gamma radiation and subsequently exposed to 0 Gy or 0.5 Gy high LET (8–10 keV/µm) proton radiation. FAECs were treated with 0 µM or 100 µM LGM2605 30 min following gamma radiation exposure. Cells were harvested 24 h post radiation exposure and evaluated for NLRP3 inflammasome activation. Fluorescent images of FAECs exposed to LGM2605 (0 µM or 100 µM) 30 min following radiation exposure and harvested at 24 h radiation exposure and incubated with anti-NLRP3 antibody (R&D Systems, Minneapolis, MN, USA). Scale bar = 20 μm (**A**); graphed representation of the data imaged for each condition (**B**). Data are presented as mean ± SEM. # shown in figures indicate significant differences from FAECs exposed to 0 Gy mixed field gamma and proton radiation and treated with 0 µM LGM2605 (#### *p* < 0.0001). Asterisks shown in figures indicate significant differences from FAECs exposed to 0.75 Gy mixed field gamma and proton radiation and treated with 0 µM LGM2605 (*** *p* < 0.001).

**Figure 9:**
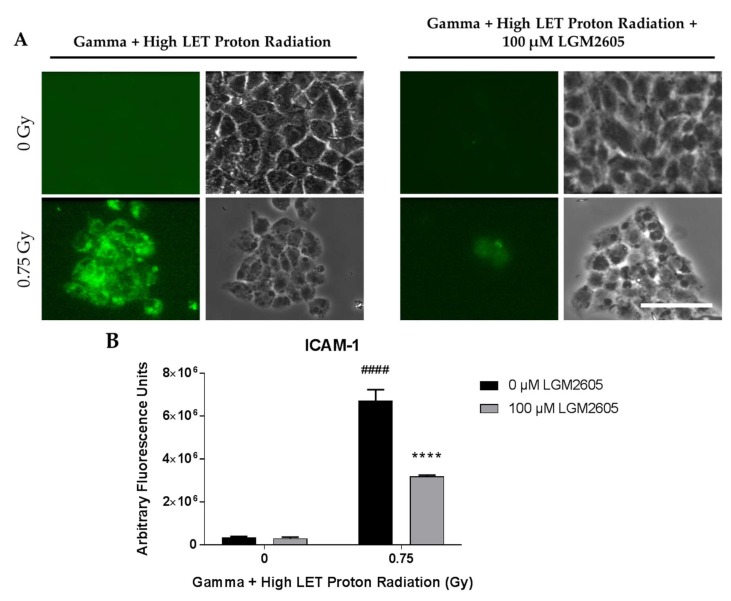
Evaluation of LGM2605 inhibition of ICAM-1 activation in in vitro lung vascular networks (flow-adapted endothelial cells) exposed to mixed field gamma and proton radiation. FAECs were exposed to 0 Gy or 0.25 Gy gamma radiation and subsequently exposed to 0 Gy or 0.5 Gy high LET (8–10 keV/µm) proton radiation. FAECs were treated with 0 µM or 100 µM LGM2605 30 min following gamma radiation exposure. Cells were harvested 24 h post radiation exposure and evaluated for ICAM-1 expression. Fluorescent images of FAECs exposed to LGM2605 (0 µM or 100 µM) 30 min following radiation exposure and harvested at 24 h radiation exposure and incubated with anti-ICAM-1 antibody (ThermoFisher Scientific, Waltham, MA, USA). Scale bar = 20 μm (**A**); graphed representation of the data imaged for each condition (**B**). Data are presented as mean ± SEM. # shown in figures indicate significant differences from FAECs exposed to 0 Gy mixed field gamma and proton radiation and treated with 0 µM LGM2605 (#### *p* < 0.0001). Asterisks shown in figures indicate significant differences from FAECs exposed to 0.75 Gy mixed field gamma and proton radiation and treated with 0 µM LGM2605 (**** *p* < 0.0001).

With high LET and a combination of high LET and gamma radiation, two observations were noted. First, a large proportion of cells detach from the glass coverslips. Approximately 34–40% of all cells remained adherent post high LET exposure, while for cells treated with both high LET and gamma (mixed radiation) 25–28% remained adherent. In contrast, low LET and gamma radiation caused minor reductions in adherence; about 92–94% of all cells remained adherent post radiation after several washes. Second, those cells that were adherent showed a “stressed appearance” in the form of altered shape, i.e., the polygonal shape was transformed into a rounded and flattened shape. In the combined exposure experiments, only about 25–28% of cells remained adherent post exposure. Overall, in the high LET and combined exposure studies, LGM2605 pretreatment did not increase the adherence of cells or protect from alteration of phenotype. Induction of the NLRP3 inflammasome and ICAM-1 expression on FAECs was determined 24 h post exposure. Mixed field gamma and proton radiation exposure induced a significant (*p* < 0.0001) robust increase in both NLRP3 and ICAM-1 expression. FAEC expression of NLRP3 and ICAM-1 was low among non-irradiated cells treated with or without 100 µM LGM2605. LGM2605 (100 µM) given during the irradiation procedure mitigated both NLRP3 and ICAM-1 induction by gamma (0.25 Gy) radiation followed by higher LET (8–10 keV/µm) proton radiation (0.5 Gy). Mixed field radiation exposure induced extensive cell death and the confluent monolayer of cells in just 24 h became patchy. The observed increases in both NLRP3 (Figure 8) and ICAM-1 (Figure 9) expression levels in FAECs exposed to gamma rays and high LET protons was significantly (*p* < 0.001) mitigated by 34.5% and 52.6%, respectively, by the action of 100 µM LGM2605.

**Figure 10:**
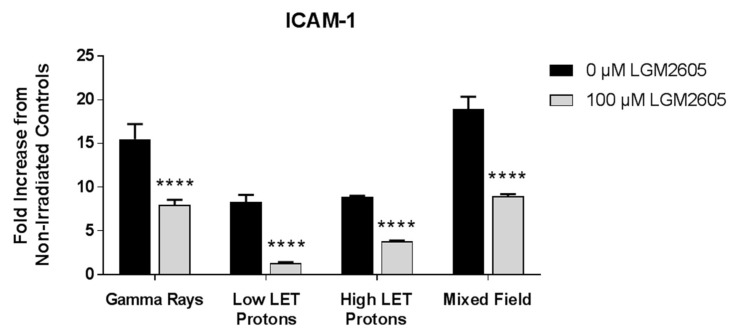
A comparative evaluation of ICAM-1 expression post radiation exposure (0.5 Gy). Fluorescent images of FAECs exposed to gamma rays, low LET protons, high LET protons, and mixed field gamma and proton radiation were quantified. Data are normalized to the respective non-irradiated controls and presented as fold increase over controls. Data are presented as mean ± SEM. Asterisks shown in figures indicate significant differences from FAECs exposed to 0.5 Gy radiation and treated with 0 µM LGM2605 (**** *p* < 0.0001).

A comparison between ICAM-1 expression levels across the various radiation exposure types following exposure to 0.5 Gy radiation shows that mixed field radiation significantly (*p* < 0.05) induced the highest expression of this adhesion molecule compared to gamma rays, low LET protons, and high LET protons (Figure 10). This indicates that for the same doses, mixed beams have higher “inflammation” potential; since these beams are representative of space relevant radiation, countermeasures need to be evaluated for their effectivity in a mixed beam environment. Levels of ICAM-1 were significantly (*p* < 0.0001) reduced for cells treated with 100 µM LGM2605 regardless of the radiation exposure type.

## 3. Discussion

In this study, the inflammatory phenotype of in vitro lung vascular networks was evaluated by monitoring the induction of cellular adhesion molecules and the NLRP3 inflammasome in FAECs exposed to gamma rays, low LET protons, high LET protons, or mixed field radiation. We have identified “detrimental biochemical signals” activated with space radiation and evaluated their mitigation by LGM2605. LGM2605 showed robust damage-mitigating properties, reducing radiation toxicity and induction of a proinflammatory phenotype. LGM2605 is a synthetic version of the bioactive lignan secoisolariciresinol diglucoside (SDG) found in the natural whole grain flaxseed, a compound that has been studied for its radioprotective and radiation mitigating properties. LGM2605 was shown to mitigate adverse chronic lung sequelae from thoracic single source radiation exposure when administered 24 h post radiation. Using both proton and gamma radiation sources to represent the solar and galactic cosmic radiation that astronauts would be exposed to in Mars and other deep space missions, we have previously reported that space relevant radiation induces inflammation and injury that is significantly mitigated with LGM2605 pretreatment of human lung slices ex vivo [22]. Among the major biological targets of space radiation are the vasculature; indeed, a high incidence of cardiovascular disease and related mortality in astronauts of past space missions has been reported, indicating a key role for space radiation in driving vascular inflammation. Limited studies that reported on vascular disease or vascular dysfunction associated with exposure to space radiation were largely confined to monitoring indices of vascular function either in astronaut populations or in intact mice [5,17,41,42]. Yet, vascular signaling events that lead to inflammation-induced damage to the vascular network have never been investigated in the context of space radiation. Research on radiation-induced damage at the cellular level has thus far focused on immune or hematopoietic cells [43,44,45], while effects of space-relevant radiation (in the form of gamma, proton, and mixed fields) on vascular endothelial cells has not been investigated to date.

**Figure 11:**
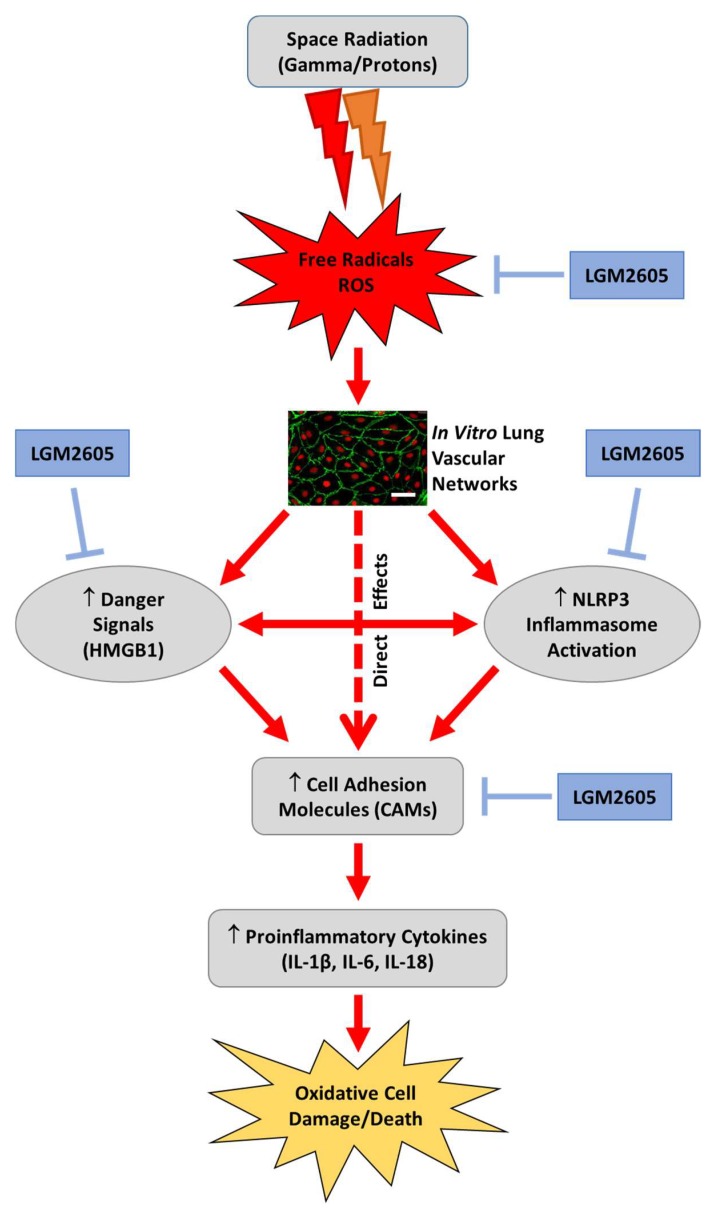
Proposed mechanism of space radiation-induced inflammation and mitigation by LGM2605 in in vitro lung vascular networks (flow-adapted endothelial cells). Space radiation exposure leads to the production of free radicals and reactive oxygen species (ROS), which ultimately activate the NLRP3 inflammasome, increase cellular adhesion molecules, and increase danger signaling molecules, such as HMGB1. LGM2605 mitigates the detrimental effects of space radiation exposure by scavenging free radicals and ROS, and subsequently blocking NLRP3 inflammasome activation and ICAM-1 expression. Scale bar = 20 μm.

Our aim was to investigate the effects of a space related radiation regimen on endothelial cell damage, loss of structure and function, and evaluate the use of the mitigating agent, LGM2605. In other inflammatory pathologies, we have found LGM2605 to abrogate proinflammatory cytokines, chemokines, and the NOD-like receptor protein 3 (NLRP3) inflammasome that are key to cell death and injury [46,47]. Specifically, prior work shows that LGM2605’s mitigating action is via three pathways: (1) direct free radical scavenging; (2) downregulation of proinflammatory cytokines; and by (3) induction of the Nrf2/ARE antioxidant pathway to boost cell antioxidant defenses (Figure 11). However, LGM2605 has not been evaluated as a countermeasure to endovascular damage post exposure to space-relevant radiation. The lung is a highly vascularized organ with >30% of the vasculature of the body in addition to being a highly radiosensitive organ. Thus, we selected to investigate the role of LGM2605 in mitigating radiation-induced lung endovascular damage by exposing an in vitro model (Scheme 1) that mimics lung vascular networks (flow-adapted endothelial cells, FAECs) to space relevant radiation.

The inflammasome is a cytosolic, multimeric signaling complex, a macromolecular protein complex that mediates proteolytic cleavage of pro-IL-1β and pro-IL-18, and induces cell death in the form of pyroptosis. It coordinates the host immune response to invading pathogens and danger signals. Certain receptors such as the nucleotide-binding oligomerization domain-like receptors (NLRs) trigger the assembly of the inflammasome in response to pathogen-associated molecular patterns (PAMPs) or danger-associated molecular patterns (DAMPs). ROS and other signals trigger inflammasome activation and pyroptosis as reviewed recently by Mathur et al. [48]. NLRP3 is known as a global sensor of PAMPs and DAMPs as a result of its ability to form the inflammasome complex in response to diverse stimuli. Activation of NLRP3 is associated with caspase-1 activation, secretion of effector cytokines IL-1β, IL-6, HMGB1, and IL-18. Exposure of tissues to radiation induces inflammatory moieties released from damaged cells that cause innate and acquired immune reactions that involve inflammasome activation [49,50,51]. Lung injury from diverse stressors including radiation exposure has been associated with inflammasome activation both by work done from our group and others [52,53]. Strategies to inhibit inflammasome activation are an active area of investigation [54]. We have identified a robust inflammasome inhibitory action of LGM2605 in an in vitro model of asbestos-induced inflammation and cell/tissue damage [46].

In light of the paucity of data on the effects of space radiation on inflammation and fibrosis, we evaluated ICAM-1 and the NLRP3 inflammasome on the vascular endothelium. We used a model that represents the vascular network in vivo. As endothelial cells in vivo are exposed to shear stress associated with blood flow, we reasoned that studies on endothelial responses to radiation ought to employ models that closely approximate the in vivo situation. Elsewhere too, reports on radiation-induced endothelial responses have shown that endothelial cells under flow respond differently to radiation than statically cultured cells [55]. This is because endothelial cells isolated from mice and kept for several passages in culture have an altered phenotype [39,56]. The lung is a highly vascularized organ with >30% of the vasculature of the body in addition to being a highly radiosensitive organ. Therefore, we employed pulmonary microvascular endothelial cells in this study. In the pulmonary vasculature, the shear stress arising from blood flow is in the range of 7–8 dyn/cm^2^; using flow chambers we thus exposed cells to similar values of shear. Our earlier reports showed that cells required a period of 24–48 h to develop a “flow adapted” phenotype and respond in a manner similar to the vasculature in vivo [29,37]. To model astronaut exposures of solar and galactic cosmic radiation, we used low and high LET proton and gamma sources and a combination of the two (mixed radiation) at radiation doses of 0.25 Gy to 1 Gy. Flow adapted pulmonary endothelial cell exposure to each of these beams showed the onset of inflammation as assessed by increased levels of ICAM-1 and the NLRP3 subunit.

Studies on radiation-induced inflammation and injury have hitherto focused primarily on immune cells. This has led to a focus on drugs that block radiation-induced immune activation. Indeed, the United States Food and Drug Administration (FDA) approved drugs, such as Neulasta or Neupogen, act (on bone marrow cells) via downregulating pathways activated by infection. This can potentially lead to an immunodeficiency among radiation-exposed subjects; besides these drugs do not act on the endothelial layer, which is the converging site of inflammation. We depart from the radiation-immune activation paradigm and focus on inflammation signals emanating from the vasculature. Blocking these signals will, we believe, lead to protection against radiation-induced inflammation and injury.

Therefore, understanding endothelial signaling pathways is crucial in the development of mitigators of radiation-induced vascular injury and dysfunction. Formulations of the phenolic SDG given after thoracic radiation mitigated radiation effects by decreasing pulmonary fibrosis, inflammation, and cytokine release, while improving blood oxygenation levels and overall mouse survival [20].

Damage to highly vascular organs, such as the lung, is often observed as a side effect of radiotherapy to treat cancer. Specifically, radiation-induced lung injury has been well characterized as a serious clinical repercussion in patients receiving unfractionated high-dose radiotherapy for lung cancer [57,58]. Acute responses to high-dose radiotherapy may include acute pneumonitis, in which a lung pathology of exuberant lung inflammation manifests as early as two weeks after radiation. Chronically, high-dose radiotherapy can lead to radiation-induced lung fibrosis, characterized by collagen deposition and scarring that manifest several months after exposure. Radiation pneumonopathy has been previously modeled in mice, which are highly susceptible to develop radiation-induced lung fibrosis [59,60,61,62]. Unlike these lung risks of high-dose low LET radiation associated with radiotherapy, space travel-associated radiation risk is the result of low-dose high-LET effects on the lung tissue. While the acute effects of space radiation on the lung are largely unknown, we have identified long-term pulmonary effects that are pathologically distinct from radiotherapy, with no noticeable lung fibrosis, but with evidence of airspace enlargement [13]. Previous work using exposure to space radiation, such as heavy ions, showed additional effects, including mutagenesis of the lung epithelium, directly from DNA damage or indirectly via production of ROS [63], and epithelial-mesenchymal transition [64,65]. Studies have shown that mitigation of oxidative damage and inflammation, intimately linked to malignant transformation, is a robust strategy to prevent carcinogenesis [46,66,67,68]. LGM2605 has been shown to reduce oxidative [22,25,27], nitrosative, and chlorination damage [69,70] as well as inflammation resulting from radiation and other stressors [24,71].

In space, radiation risk stems from exposure to GCR and SPE, which contain high-energy nuclei (HZE) with an electric charge higher than +2 (such as ^56^Fe and ^28^Si ions) and high- and medium-energy protons (H^+^). Little is known about the lung’s response to GCR and SPE exposures whose high linear energy transfer (LET) components penetrate deeply into exposed tissues and produce secondary radiations. There are important, but limited, studies addressing radiation to the lung [63,72,73,74,75,76]. In contrast, there are extensive studies on the effects of high-dose terrestrial, low LET radiation such as gamma- and X-rays on various tissues, including the lung. Given their distinct physical properties, the pathobiology of lung damage induced by low-dose space-relevant radiation exposure cannot be extrapolated from that induced by high-dose terrestrial radiation.

Our findings support the potential use of LGM2605 as a countermeasure to endovascular damage post exposure to space-relevant radiation. Using a model that mimics the lung vascular system (flow-adapted endothelial cells; FAECs) exposed to space-relevant radiation (low-dose gamma rays, low LET protons, high LET protons, and mixed field radiation), our results provide robust evidence that this agent can be an effective radiation countermeasure agent. In summary, we identified a significant reduction of inflammatory and oxidative changes in irradiated FAECs when LGM2605 was administered within 30 min of radiation exposure. Importantly, the damage induced by the type of radiation (gamma rays, protons, or mixed fields) or the level of radiation (low or high LET) was equally mitigated by the addition of LGM2605. We conclude that this agent, LGM2605, is a likely candidate as a countermeasure to reduce tissue damage from space-relevant radiation exposure.

## 4. Materials and Methods

### 4.1. Cells and Culture Media

Isolation and culture of pulmonary microvascular endothelial cells have been described previously [29,37]. Briefly, endothelial cells were grown in Dulbecco’s low glucose modified Eagle’s medium supplemented with 10% fetal bovine serum (FBS), nonessential amino acids, and penicillin/streptomycin. Endothelial cells were maintained under static culture conditions for several passages before being subjected to flow.

### 4.2. Exposure of Cells to Shear Stress

Cells were seeded at 8000 cells/cm^2^ in medium comprised of low glucose DMEM supplemented with 10% FBS and essential amino acids. The cells reached confluency after 24 h. Cells were lightly washed with medium without FBS and fitted into the chamber slot. A parallel plate confocal imaging chamber (Warner Instruments, LLC, Hamden, CT, USA) was used to adapt endothelial cells to flow on coverslips as reported earlier [29,31]. The chamber consisted of two metal circular plates that encased silicone precut gaskets. This created a hollow slot in the center that is fitted with a coverslip containing cells to create a rectangular flow channel (125 µm high, 2 cm wide, and 2 cm long). Inlet and outlet ports on the steel plate were connected to a pump and a dual reservoir to facilitate flow of medium in a pulsatile manner (to recreate the cardiac rhythm) into the system (see Scheme 1). Cells were grown in gelatin-coated cell culture dishes until confluence after which these cells were trypsinized and replated on coverslips pre-coated with fibronectin. Both gelatin and fibronectin serve to replicate the basement matrix in vivo. The basement matrix is the interface between the endothelial cells and adjacent tissue. For gelatin coating, a minimum volume of 1% gelatin was added to the cell culture dishes for 300 min at 37 °C after which cells were plated. Once cells were confluent these were removed and allowed to grow on glass coverslips. To facilitate better adhesion under flow, these coverslips were coated with fibronectin (1 mg/mL in a dilution ratio of 1:100) for 45 min at room temperature, after which excess fibronectin was removed by aspiration. Pulmonary microvascular endothelial cells grown to confluency on fibronectin coated glass coverslips (2 cm × 2 cm) were then fitted into the chamber slot, where plastic coverslips were used to seal the flow chambers. With this apparatus, cells were subjected to shear stress for 24 h at 7 dyn/cm^2^.

### 4.3. Radiation Exposure

Flow-adapted endothelial cells were exposed to gamma radiation with a Shepherd Mark 1 ^137^Cs irradiator delivering a dose of 1.0 Gy/minute. FAECs were proton irradiated with protons in the Robert’s Proton Therapy facility at the University of Pennsylvania. With the intention of increasing the LET of the dose delivered to the cells, the geometry shown in Figure 5 was used. Six cell dishes were placed in a central holder within a cylindrical phantom of solid water of 20 cm of diameter. Solid water phantoms are made of epoxy resins and powders to control density and radiation properties of the phantom and are traditionally used for dosimetry purposes. A field size was selected to ensure that all dishes were simultaneously exposed. Cells were exposed to 0.25 and 0.5 Gy using thirteen mono-energetic beams placed every 15 degrees over a total of 180-degree arc. For every angle, a mono-energetic beam with energy 117.14 MeV (10.1 cm range in water) was used to target the central plane of the dishes. This implied that every beam had its distal 90% dose placed at the central plane of the phantom, and laterally extended to cover the diameter of the dish 1 cm on each side to ensure dose homogeneity across the fluid contained in the dishes. The resultant dose distribution from the sum of the dose of each field resulted in a homogeneous dose to the target. The dose to the target was optimized based on multi-field dose optimization (MFO) mode. Also, as all beams stopped within the target, the higher LET components of each beam were placed within the target. Both dose and dose-average LET distributions are shown in Figure 5.

### 4.4. LGM2605 Treatment

Synthesis of secoisolariciresinol diglucoside has been previously described [25]. Briefly, secoisolariciresinol diglucosides (*S*,*S*)-SDG (the major isomer in whole grain flaxseed) and (*R*,*R*)-SDG (the minor isomer in whole grain flaxseed) were synthesized from vanillin via secoisolariciresinol and a glucosyl donor (perbenzoyl-protected trichloroacetimidate under the influence of TMSOTf) through a concise route that involved chromatographic separation of diastereomeric diglucoside derivatives. Synthetic SDG (LGM2605) was reconstituted to a stock concentration of 10 mM using cell culture phosphate buffered saline without calcium and magnesium. Based on our previous work [22,27], FAECs were treated with 100 µM LGM2605 30 min following radiation exposure.

### 4.5. Determination of ICAM-1 and NLRP3 Expression

Levels of ICAM-1 and NLRP3 were determined using an anti-ICAM mouse-monoclonal antibody at 1:250 (ThermoFisher Scientific, Waltham, MA, USA) and a rabbit polyclonal anti-NLRP3 antibody at 1:200 (R&D Systems, Minneapolis, MN, USA). Secondary antibodies tagged to fluorescent Alexa 488 (green) were used at 1:200 (ThermoFisher Scientific, Waltham, MA, USA). Flow-adapted endothelial cells were exposed to gamma, proton, or mixed field radiation and treated with LGM2605 30 min post radiation exposure. Cells were then fixed with 4% paraformaldehyde 24 h post radiation exposure and kept at 4 °C. Cells were permeabilized and immunostained for ICAM-1 and NLRP3 by using anti-ICAM-1 and anti-NLRP3 antibodies. After washing cells several times and labeling with secondary antibodies, slides were dried and imaged. Images were acquired at 500 ms exposure on a Nikon TMD epifluorescence microscope (Nikon Diaphot TMD, Melville, NY, USA), equipped with a Hamamatsu ORCA-100 digital camera (Hamamatsu Photonics K.K., Hamamatsu City, Japan) and MetaMorph imaging software (Molecular Devices, Downington, PA, USA). The green fluorescent signal in these images represents the amount of ICAM-1 or NLRP3 in these FAECs. The intensity of the fluorescent signal was quantified by integrating the fluorescence of all cells within the entire field and normalizing to the equal field area using Metamorph Imaging Software (Molecular Devices, Downington, PA, USA) and ImageJ software (Fiji Version, National Institutes of Health, Bethesda, MD, USA). The background was subtracted to obtain “corrected” intensity values. All fluorescence images were acquired at the same exposure and offset acquisition settings. At least 6 fields were imaged and analyzed per condition/treatment and data from 3–4 independent or separate experiments were averaged to obtain the final results. Scale bar = 20 μm.

### 4.6. RNA Isolation and Gene Expression Analysis

Total RNA was isolated from flow-adapted endothelial cells using a commercially available kit, RNeasy Plus Mini Kit, supplied by Qiagen (Valencia, CA, USA), as previously described [46,71]. Total RNA concentrations and 260/280 ratios were determined using a NanoDrop 2000 apparatus (ThermoFisher Scientific, Waltham, MA, USA). Reverse transcription of RNA to cDNA (1.8 µg of total RNA) was then performed on an Applied Biosystems Veriti^®^ Thermal Cycler (ThermoFisher Scientific, Waltham, MA, USA) using the high capacity RNA to cDNA kit supplied by Applied Biosystems followed by Quantitative Polymerase Chain Reaction (qPCR) analysis using TaqMan^®^ Probe-Based Gene Expression Assays supplied by Applied Biosystems, Life Technologies (Carlsbad, CA, USA). Individual TaqMan gene expression assays were selected for *heme oxygenase-1* (*HO-1*), *NADPH: quinone oxidoreductase-1* (*NQO1*), and *glutathione S-transferase Mu 1* (*GSTM1*). Quantitative real-time PCR was performed using 50 ng of cDNA per reaction well on a StepOnePlus™ Real-Time PCR System (Applied Biosystems, Life Technologies, Carlsbad, CA, USA). Gene expression data were normalized to both 18S rRNA (Figure 1) and GAPDH (data not shown) housekeeping genes. Relative quantification was determined calibrated to the FAECs exposed to 0 Gy gamma radiation and treated with 0 µM LGM2605 according to the ΔΔCT method as previously described [20].

### 4.7. Statistical Analysis

All data were analyzed using two-way analysis of variance (ANOVA) to test for the main effects of radiation exposure and LGM2605 treatment, along with the interaction between these variables, on study outcome measures. Post-tests (Tukey’s multiple comparisons tests) were conducted analyzing significant differences between radiation exposure groups (non-irradiated versus irradiated) and among treatment groups (no LGM2605 versus LGM2605). Statistically significant differences were determined using GraphPad Prism version 6.00 for Windows, GraphPad Software, La Jolla, CA, USA, www.graphpad.com. Results are reported as the mean ± the standard error of the mean (SEM). Levels of target gene mRNA are reported as the mean fold change from FAECs exposed to 0 Gy radiation and treated with 0 µM LGM2605 ± SEM. Statistically significant differences were determined at *p*-value of 0.05. # shown in figures indicate significant differences between radiation exposure groups (non-irradiated versus irradiated) (# *p* < 0.05, ## *p* < 0.01, ### *p* < 0.001 and #### *p* < 0.0001). Asterisks shown in figures indicate significant differences between treatment groups (no LGM2605 versus LGM2605) (* *p* < 0.05, ** *p* < 0.01, *** *p* < 0.001 and **** *p* < 0.0001).

## 5. Conclusions

We have shown space radiation-induced inflammatory phenotype, inflammasome activation, and damage post radiation. Additionally, we have identified the ability of LGM2605 to inhibit the inflammatory phenotype in in vitro lung vascular networks exposed to space radiation.

## Abbreviations

ACS	active chlorine species
ARE	antioxidant response element
CTL	Control
DAMP	danger-associated molecular pattern
EC	endothelial cell
ELISA	enzyme-linked immunosorbent assay
FAEC FLC	flow-adapted endothelial cell flaxseed lignan component
FS	Flaxseed
GCR	galactic cosmic ray
GSTM1	glutathione S-transferase mu 1
HO-1	heme oxygenase-1
huPCLS	human precision-cut lung sections
HZE	high-energy nuclei
ICAM-1	Intercellular cell adhesion molecule-1
IL-1β	interleukin-1β
IL-6	interleukin-6
LGM2605	synthetic secoisolariciresinol diglucoside
MFO	multi-field dose optimization
NLR	nucleotide-binding oligomerization domain-like receptor
NLRP3	NOD-like receptor protein 3
Nrf2	nuclear factor (erythroid-derived 2)-like 2
Nqo1	NADPH: quinone oxidoreductase-1
PAMP	pathogen-associated molecular pattern
PBS	phosphate-buffered saline
qPCR	quantitative polymerase chain reaction
RNS	reactive nitrogen species
ROS	reactive oxygen species
SDG	secoisolariciresinol diglucoside
SEP	solar energetic particle
SOBP	spread of Bragg peak
SPE	solar particle event
TNFα	tumor necrosis factor alpha
TRR	total radiation risk
